# Effect of soaking in plasma-activated liquids (PALs) on heavy metals and other physicochemical properties of contaminated rice

**DOI:** 10.1016/j.fochx.2024.101788

**Published:** 2024-08-30

**Authors:** Shahnaz Bohlooli, Yousef Ramezan, Fatemeh Esfarjani, Hedayat Hosseini, Soheyl Eskandari

**Affiliations:** a Department of Food Science and Technology, Faculty of Pharmacy, Tehran Medical Sciences, Islamic Azad University, Tehran, Iran; b Nutrition & Food Sciences Research Center, Tehran Medical Sciences, Islamic Azad University, Tehran, Iran; c Research Department of Food and Nutrition Policy and Planning, Faculty of Nutrition Sciences and Food Technology, National Nutrition & Food Technology Research Institute (NNFTRI), Shahid Beheshti University of Medical Sciences, Tehran, Iran; d Department of Food Science and Technology, National Nutrition and Food Technology Research Institute, Faculty of Nutrition Science and Food Technology, Shahid Beheshti University of Medical Sciences, Tehran, Iran; e Food Safety Research Center, Shahid Beheshti University of Medical Sciences, Tehran, Iran; f Food and Drug Laboratory Research Center (FDLRC), Food and Drug Administration (IR-FDA), Ministry of Health and Medical Education (MOH+ME), Enghelab St., Fakhr-e Razi St., Tehran, Iran; g Department of Community Nutrition, School of Nutritional Sciences and Dietetics, Tehran University of Medical Sciences, 1416643931 Tehran, Iran

**Keywords:** Heavy metals, Non-thermal plasma, Texture profile analysis, Thermal properties

## Abstract

In this study, plasma-activated liquids (PALs) were produced by a cold plasma gliding arc device at two different exposure times (7.5 and 15 min) and compared with deionized water (DW) as a control. The results showed that the amount of arsenic (As: 98 %), cadmium (Cd: 93 %), and lead (Pb: 93.3 %) were significantly decreased in all samples after soaking in PALs and DW than raw rice (*p* *<* *0.05*). However, 15-min PALs were more successful. All soaked samples did not exceed the maximum residue limits (MRLs). A softer and easier chewing texture was observed for rice samples soaked in PALs than the sample soaked in DW. The samples treated with PALs also showed a lower gelatinization temperature and enthalpy. The color parameters and microstructure of rice samples were affected by treatment with PALs. Therefore, soaking rice in PALs before cooking can be considered an effective method to reduce the heavy metals in rice.

## Highlights

• Plasma-activated liquids (PALs) decreased selected heavy metals in rice.
• PALs activated for 15 min were more successful in heavy metal decontamination.
• The texture, microstructure, and thermal properties of rice were changed by PALs.
• Total color differences were not distinct after soaking rice in PALs.

## 1 Introduction

Rice (*Oryza sativa* L.) is the second highest-produced grain worldwide and stands as a prominent food crop and a fundamental dietary staple for more than half of the global population (Mohammadi et al., 2021). However, the quality and safety of rice products can be affected by contamination with heavy metals. The presence of heavy metals in the rice is attributed to different environmental and technical factors, such as the improper use of pesticides and fertilizers containing heavy metals and the contamination of the water sources used for irrigation (Abu-Almaaly, 2020). The presence of heavy metals such as As, Cd, and Pb in rice can be very harmful to consumers because these elements are highly toxic to humans and can contribute to a variety of serious adverse health effects in humans and living bodies (Khanom & Hayashi, 2021; Zhai et al., 2019; Zhang et al., 2017). In this regard, various methods have been used to remove or reduce heavy metals from the contaminated rice. For example, in this case, it was reported that these heavy metals could be removed to the greatest extent if rice was washed and cooked in abundant amounts of water (Mihucz et al., 2007). In another study, soaking in the presence of NaCl or sour lemon peel as a biosorbent was used as an efficient method to remove heavy metals from *Oryza sativa* rice from Astaneh Ashrafieh, Gillan province, in the north of Iran (Razafsha et al., 2016). Some studies also used microbial fermentation to reduce Cd levels in the rice (Zhai et al., 2019). The soaking at an appropriate time and temperature was also suggested as an effective approach to reduce the amount of Cd and Pb in the rice, which reduces public exposure to these toxic elements (Al-Naimi & Al-Ghouti, 2020). Plasma-activated water (PAW) or plasma-activated liquids (PALs) are prepared by treating water or other liquids with non-thermal cold atmospheric plasma using controllable parameters such as voltage, temperature, pulse, and carrier gas (Chumsri et al., 2022; Soni et al., 2021). PAW is a mixture of highly biochemically reactive solutions that has an acidic condition, attributing changes in oxidation-reduction potential (ORP), electrical conductivity (EC), and the formation of reactive oxygen and nitrogen species (ROS and RNS) (Hou et al., 2021). In the food industry, PAW serves a variety of purposes, including disinfecting, inactive food spoilage microorganisms and foodborne pathogens (Han et al., 2020; Niu et al., 2024) and can also be used to enhance the growth, sprouting, and germination of plant seeds (Chuea-uan et al., 2024; Rashid et al., 2022). In a study conducted by Hou et al. (2021), PAW was used to reduce the accumulation of heavy metals in water spinach. In another study conducted by Qu et al. (2013), pulsed corona-discharged plasma combined with activated carbon was successfully used to remove cadmium ions from the water solution. The PAW was also used by Kang et al. (2022) to inactivate the indigenous mesophilic aerobic bacteria and *coliforms* on steamed rice cake. In addition to the application as a method to reduce the contamination of food products, it was reported that plasma processing can be used as a treatment to improve their technological functionalities. For example, it was reported by Thirumdas et al. (2015) that the cooking quality of the basmati rice was improved by treating it with low-temperature plasma. Sarangapani et al. (2016) also investigated whether the flour/gel hydration properties of the parboiled rice flour were improved by low-pressure plasma. It was also observed that PAW could improve the resistant starch content in the starch industry and the multi-structural modification of starches (Yan et al., 2020). Other solutions, such as normal saline or medium, instead of water, can be used to prepare plasma-activated normal saline (PAS) or plasma-activated medium for application on microbial decontamination and anticancer effects (Huang et al., 2023).

Therefore, this is the first study on using the green technology of PAW and PAS to reduce the amount of heavy metals such as As, Cd, and Pb in contaminated rice. Moreover, the effect of these treatments on the physical, thermal, textural, and microstructural attributes of the rice samples was determined.

## 2 Materials and methods

### 2.1 Sample collection

From 2022 to 2023, imported Indian rice was randomly sampled from imported rice in Tehran customs as part of the Iran Food and Drug Administration (IFDA) inspection and monitoring programs. Authorized and trained IFDA inspectors collected 300 representative samples (not less than 1 kg from each batch) following CODEX Alimentarius Commission general guidelines on sampling (FAO/WHO, 2004). After collection, samples were transported immediately to the IFDA laboratories campus (Tehran, Iran) in suitable dry containers and stored the samples at 25 °C until analysis.

### 2.2 Sample preparation

**Fig. 1:**
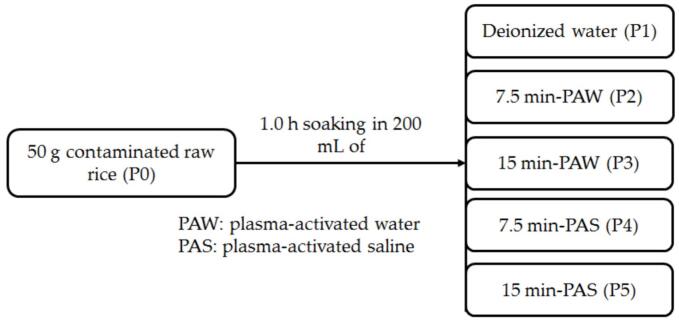
Different rice samples produced in this study.

For the different sample preparations, 50 g of rice was weighed and washed three times with deionized water. Six different samples were prepared for this study. Sample P0 was the contaminated raw rice, and the sample (P1) was prepared by soaking the rice for 1 h, followed by washing in 200 mL of deionized water. The PAW and PAS were prepared by 7.5 and 15 min of treatment of 200 mL of water without and with 1 % NaCl at a power of 100 W using a cold atmospheric gliding arc plasma device (GA 500, Plasma Clean- Satia Company, Iran). This was followed by adding 50 g of the washed rice to these PAW samples, and the rice was soaked for 1 h in 7.5- and 15-min PAW to prepare samples P2 and P3. To study the effect of salt (NaCl), 50 g of the washed rice was added to 200 mL of 7.5- and 15-min PAS in the presence of 1 % NaCl and soaked for 1 h to prepare samples P4 and P5. Table 1 and Fig. 1 present the characteristics of the samples obtained in the present study.

**Table 1:** Different samples prepared in the present study.

Sample	Treatment
P0	Raw rice without washing
P1 (control)	1.0 h soaking in deionized water
P2	1.0 h soaking in (7.5 min) plasma-activated water
P3	1.0 h soaking in (15 min) plasma-activated water
P4	1.0 h soaking in (7.5 min) plasma-activated saline with 1 % NaCl
P5	1.0 h soaking in (15 min) plasma-activated saline with 1 % NaCl

### 2.3 pH measurement

The pH of the PAW and PAS samples with and without rice was determined using a digital pH meter (Model MW151MAX, pH/ORP/Temp Meter, Europe). The ORP of the activated water samples and the activated water samples in the presence of rice after 1 h of soaking was determined using the above-mentioned pH meter apparatus.

### 2.4 Moisture content

The moisture content of different rice samples was measured using a humidity meter (Humidity Meter Model MA35 Sartorius, Germany).

### 2.5 Determination of heavy metals

The inductively coupled plasma mass spectrometry (ICP-MS) method using a Perkin Elmer ELAN 6100 DRC-e ICP-MS system was used to analyze the concentrations of elements, including As, Pb, and Cd, in the rice samples according to the method described by Hoseini et al. (2023). Initially, the heat-block-assisted acid digestion method used 65 % nitric acid and 37 % hydrogen peroxide to digest the rice samples. After that, the digested samples were diluted with deionized water, filtered using acid-resistant filter paper, and then stored for the ICP-MS test (Hoseini et al., 2023).

### 2.6 Textural properties

Texture profile analysis (TPA) of the rice grains (with an average length of 0.9–1.0 cm) was performed using a texture analyzer (TAXT plus, stable-micro system, United Kingdom) with a two-cycle compression. A cylindrical probe P/36 R was used to compress rice grains with a test speed of 5 mm/min. The instrument was calibrated with a 50 kg load cell. The deformation ratio was set at 50 %. Fifteen pairs of grains were measured for each sample (Chen et al., 2012).

### 2.7 Thermal properties

The thermal behaviors of the rice samples were studied using differential scanning calorimetry (DSC) (Model Differential Thermal Analyzer, DSC 214 Polyma Differential Scanning Calorimeter, NETZSCH-Gerätebau GmbH, Germany). Each sample was weighted directly in a DSC pan and was then hermetically sealed and allowed to stand for 1 h before thermal analysis. Thermal scanning was undertaken from 4 °C to 150 °C at a heating rate of 10 °C/min. The gelatinization onset (To), peak (Tp), and conclusion (Tc) temperatures and the transition enthalpy (ΔH) were determined and reported (Lee et al., 2019).

### 2.8 Color parameters

The color parameters of the samples, including L* or lightness (black to white), a* (greenness to redness), and b* (blueness to yellowness), were measured using a Hunter Lab Colorimeter model STB2000 Faratel (Color Flex EZ, Hunter Associates Laboratory 11,491 Sunset Hills Road Reston, Virginia 20,190 USA) (Martins et al., 2021). From these parameters, the total color difference (∆E) of the treated samples and the untreated sample, which is here as a reference, was calculated using Eq. (1). Also, the whiteness index (WI) and browning index (BI) were measured according to Eqs. (2), (3), (4), respectively (Afshar et al., 2022).

(1) $$∆E=\sqrt{∆L^{2}+∆a^{2}+∆b^{2}}$$

(2) $$\mathrm{WI}=100-\sqrt{{\left(100-L^{*}\right)}^{2}+a^{*2}+b^{*2}}$$

(3) $$\mathrm{BI}=\frac{\left(x-0.31\right)}{0.17}\times 100$$

(4) $$\text{Where}\;x=\frac{\left(a^{*}+1.75L^{*}\right)}{5.645L^{*}+a^{*}-3.012b^{*}}$$

### 2.9 Microstructure

The microstructure of different rice samples was studied by using a scanning electron microscope (SEM, VEGA/TESCAN, Pennsylvania, USA). For this purpose, the samples were coated with a thin layer of gold before taking the SEM micrographs.

### 2.10 Statistical analysis

All the experiments were performed in triplicate orders. The results were statistically analyzed using a one-way ANOVA using SPSS software version 19. Duncan's multiple range post-hoc test was used to determine the significant differences (*p* *<* *0.05*).

## 3 Results and discussion

### 3.1 pH, ORP, and moisture content

The pH and ORP values of the PAW and PAS with and without rice are presented in Table 2. In all samples, an increase in plasma exposure time resulted in a decrease in pH and an increase in ORP. Accordingly, the 7.5-min PALs had a higher pH and lower ORP than the 15-min PALs. The pH and ORP values were also affected by the addition of NaCl. The PAS samples had a lower pH and ORP than the PAW samples. In agreement with these findings, it was reported that the ORP and pH values of the reverse osmotic (RO) water decreased and increased, respectively, with increasing plasma exposure time (Hou et al., 2021). It was investigated that the ORP of plasma water was increased with longer exposure times to the plasma treatment, indicating a higher generation of reactive oxygen species (Joshi et al., 2018). A lower pH was reported for beef after thawing with PAW due to the presence of a large amount of active substances (Wang et al., 2024).

**Table 2:** The pH, ORP, and moisture content of different rice samples before adding and after 1.0 h soaking in deionized water and PALs.

Sample	pH		ORP (mV)		Rice moisture content (%)
	PALs	PALs + rice	PALs	PALs + rice	
P1	–	6.92 ± 0.01^e^	–	–	36.42 ± 0.24^c^
P2	4.14 ± 0.01^d^	5.85 ± 0.01^d^	362.5 ± 1.00^b^	234 ± 1.00^a^	40.22 ± 0.16^b^
P3	3.88 ± 0.01^b^	4.37 ± 0.01^b^	477 ± 1.00^d^	293 ± 1.00^b^	41.30 ± 0.35^a^
P4	3.92 ± 0.01^c^	4.93 ± 0.01^c^	334 ± 1.00^a^	308.3 ± 1.00^c^	40.13 ± 0.02^b^
P5	3.72 ± 0.01^a^	4.13 ± 0.01^a^	368.6 ± 1.00^c^	308.7 ± 1.00^c^	41.22 ± 0.10^a^

Results are presented as a mean value ± standard deviation (*n* = 3). Means with different letters in the same column are significantly different (*p* *<* *0.05*). (P1: Control (deionized water); P2: PAW (7.5 min); P3: PAW (15 min); P4: PAS (7.5 min); P5: PAS (15 min)). Abbreviations: PALs: plasma-activated liquids; ORP: oxidation-reduction potential; PAW: plasma-activated water; PAS: plasma-activated saline.

As shown in Table 2, significant differences were observed in the moisture content of the rice samples. Results showed that the samples that were soaked in the PALs had a significantly higher moisture content compared to the rice sample soaked in the non-treated deionized water (*p* *<* *0.05*). The samples soaked in 15-min PALs also had a slightly higher moisture content than those soaked in 7.5-min PALs. The addition of NaCl also did not have a significant effect on the rice moisture content (*p* *>* *0.05*). The higher moisture content of the samples soaked in the PALs can be due to their higher ability to penetrate the rice structure. In accordance with our results, it was reported that the water uptake of Chinese milled rice (Liu et al., 2021) and basmati rice (Thirumdas et al., 2016) was increased by treating them with cold plasma.

### 3.2 Heavy metals content

The maximum residue limits (MRLs) of Cd, Pb, and AS in rice have been established at 0.06, 0.15, and 0.15 mg/kg by the Iran National Standard Organization (INSO, 2021). The MRLs have been set by JECFA (WHO/FAO) for Cd, Pb, and AS are 0.4, 0.2, and 0.2 mg/kg, respectively (FAO/WHO, 2004). The amounts of different heavy metals, including As, Cd, and Pb, in the rice samples are shown in Table 3.

**Table 3:** The heavy metals concentrations (μg/kg) in treated and untreated rice samples by PALs.

Sample	As	Cd	Pb
P0	4200 ± 100^b^	200 ± 1.00 ^b^	1000 ± 100 ^b^
P1	147.900 ± 1.00^aA^	28.70 ± 1.00^aA^	104.20 ± 1.00^aA^
P2	104.66 ± 1.20^aB^	24.20 ± 1.00^aAB^	67.10 ± 1.00^aC^
P3	80.00 ± 1.00^aD^	20.00 ± 1.00^aB^	82.20 ± 1.00^aB^
P4	92.30 ± 1.00^aC^	14.60 ± 1.00^aC^	100.00 ± 1.00^aA^
P5	81.200 ± 1.00^aD^	24.10 ± 1.00^aB^	71.80 ± 1.00^aC^

Results are presented as a mean value ± standard deviation (n = 3). Means with different lowercase letters in the same column are significantly different (All samples; P0-P5) (*p* *<* *0.05*). Means with different Uppercase letters in the same column are significantly different (soaked in deionized water and Plasma-activated liquids; P1-P5) (*p* *<* *0.05*). (P0: Contaminated raw rice, P1: Control (deionized water); P2: PAW (7.5 min); P3: PAW (15 min); P4: PAS (7.5 min); P5: PAS (15 min)). Abbreviations: PALs: plasma-activated liquids; PAW: plasma-activated water; PAS: plasma-activated saline.

According to the results, the heavy metal content in raw rice exceeded the MRLs. However, after 3 times of washing and 1 h of soaking in DW and PALs, the amount of selected heavy metals significantly decreased in all soaked samples. The results indicated that the PALs were more efficient in decreasing the heavy metal content in rice. All activated liquids, especially those that were activated for a longer time (samples P3 and P5), were the most successful in this regard, and the reduction rates were higher than the rice sample soaked in DW. All samples, after soaking, were safe to cook and consume (Table 3).

The results showed that the soaking of rice in DW and PALs with or without NaCl addition significantly decreased the amount of As, Cd, and Pb in the rice samples (*p* *<* *0.05*). The lowest level of As (80 μg/kg) was observed in sample P3, which was soaked in 15-min PAW. The highest amount of As (4200 μg/kg) was found in the sample P0 (raw rice). Moreover, the results showed that the level of As in sample P3 was lower than P2, and the level of As in sample P5 was also lower than P4, suggesting that the higher plasma activation time can be more effective in As reduction in the rice samples. The addition of NaCl to the 15-min PAS also resulted in a higher reduction of As. The lowest amount of Cd (14.60 μg/kg) was observed in sample P5, which was soaked in the 15-min PAS. The highest amount of Cd (200 μg/kg) was determined in the sample P0. The lowest amount of Pb (67.1 μg/kg) was found in sample P3, which was soaked in 15-min PAW. The highest amount of Pb (1000 μg/kg) was also determined in the sample P0.

It should be noted that in comparison of PALs with DW, PLAs significantly reduced selected heavy metals in rice compared to DW, except for samples P1 and P2 for Cd and P1 and P4 for Pb (*p* *<* *0.05*).

Generally, these findings suggest that PALs can be used as an effective method to reduce heavy metals in rice, which ultimately improves the safety of the product for humans. The reduction of heavy metals in rice after soaking in PALs can be related to the changes in water pH and ORP after treatment with plasma, which can affect the solubility of heavy metals in the rice samples (Thakulla & Fisher, 2023). For example, it was reported that the soaking of rice in water with a lower pH can increase the leaching of metals from rice starch due to the competition between metals and excess H^+^ ions for binding to the carboxyl group of starch as a metal binding site (Al-Naimi & Al-Ghouti, 2020). The effect of soaking solution pH on the removal of heavy metals was also reported for rice bran by Mohammadi et al. (2021).

### 3.3 Textural attributes

The TPA analysis was employed to characterize the textural attributes of the rice samples, and the results are presented in Table 4. The results indicated that the effect of soaking in PALs did not have a significant effect on the adhesiveness, fracturability, and springiness of the rice samples (*p* *>* *0.05*). The TPA results also indicated that the highest chewiness (258.52), cohesiveness (0.38), gumminess (897.2), and hardness (2716 g) were related to the control sample (P1), and the soaking in PALs significantly decreased these parameters (*p* *<* *0.05*). The lowest chewiness (28.94) and cohesiveness (0.20) were observed in sample P2, where the rice sample was soaked in 7.5-min PAW. The lowest gumminess (39.7) and hardness (360.7 g) were determined in sample P5, in which the rice sample was soaked in 15-min PAS. According to these findings, it seems that the soaking in PALs resulted in rice with a softer texture and an easier texture to chew. In accordance with our findings, Chen et al. (2012) also reported an increase in adhesiveness and a decrease in the hardness of brown rice, as a result of treatment with plasma. Therefore, it was suggested that the cold plasma can be considered an efficient pre-treatment to reduce the cooking time required for rice (Lee et al., 2019). The effect of PAW on the textural properties of rice can be related to its effect on the rice microstructure, hydration process, and leaching of starch components in different proportions of amylose and amylopectin, resulting in a texture with a lower hardness (Sarangapani et al., 2015; Thirumdas et al., 2015; Zhang et al., 2015). In the case of basmati rice, it was reported that the low-temperature plasma processing can decrease the hardness, which can be due to the leaching of solids from the rice grains (Thirumdas et al., 2015).

**Table 4:** The texture profile analysis of different rice samples treated and untreated by PALs.

Sample	Adhesiveness (g.s)	Chewiness	Cohesiveness	Fracturability (g)	Gumminess	Hardness (g)	Resilience	Springiness
P1	−1.85 ± 0.02 ^a^	258.52 ± 20.38 ^a^	0.38 ± 0.01 ^a^	237.91 ± 26.28 ^a^	897.2 ± 127.4^a^	2716 ± 147.3^a^	0.31 ± 0.01^a^	0.34 ± 0.05^a^
P2	−3.35 ± 0.74 ^a^	28.94 ± 3.56 ^c^	0.20 ± 0.01^b^	140.28 ± 82.42 ^a^	114.7 ± 18.5^b^	665.5 ± 46.1^b^	0.17 ± 0.01^a^	0.34 ± 0.02^a^
P3	−2.02 ± 0.01 ^a^	49.20 ± 5.06 ^b^	0.36 ± 0.03 ^a^	53.98 ± 5.59 ^a^	56.2 ± 9.5^b^	1890 ± 41.2^b^	0.28 ± 0.11^a^	0.26 ± 0.07^a^
P4	−1.75 ± 0.22 ^a^	52.15 ± 5.79 ^b^	0.24 ± 0.03 ^b^	95.52 ± 20.15 ^a^	125.7 ± 52.5^b^	687.1 ± 72.2^b^	0.29 ± 0.01^a^	0.37 ± 0.10^a^
P5	−2.58 ± 0.51 ^a^	41.60 ± 3.69 ^bc^	0.34 ± 0.01 ^a^	197.10 ± 25.40 ^a^	39.7 ± 18.5^b^	360.7 ± 34.6^b^	0.27 ± 0.04^a^	0.29 ± 0.09^a^

Results are presented as a mean value ± standard deviation (n = 3). Means with different letters in the same column are significantly different (*p* *<* *0.05*). (P1: Control (deionized water); P2: PAW (7.5 min); P3: PAW (15 min); P4: PAS (7.5 min); P5: PAS (15 min)). Abbreviations: PALs: plasma-activated liquids; PAW: plasma-activated water; PAS: plasma-activated saline.

### 3.4 Thermal properties

The thermal properties of different rice samples including gelatinization onset (To), peak (Tp), and conclusion (Tc) temperatures, and the transition enthalpy (ΔH), were determined and the results are shown in Table 5. The gelatinization temperature was determined to study the required energy to start the starch gelatinization (Liu et al., 2021). The results indicated that the thermal properties of the samples soaked in PAL were significantly different compared to the control sample (*p* *<* *0.05*). The onset temperature of the samples soaked in PALs was lower than the control sample. Samples treated with 7.5-min PALs also had a lower onset temperature compared with the samples soaked in 15-min PALs. Moreover, the results showed that the addition of NaCl also changed the onset temperature, especially in the samples soaked in 15-min PAS. The lowest peak temperature was found in sample P5, which was soaked in the 15-min PAS (*p* *<* *0.05*). Generally, the samples soaked in 15-min PALs had a lower peak temperature compared to those samples soaked in 7.5-min PALs. A higher conclusion temperature was observed for samples treated with 7.5-min PALs, and a lower conclusion temperature was observed for rice samples soaked in 15-min PAW than the control sample (*p* *<* *0.05*). The lower peak temperature for the samples soaked in PALs can be due to the de-crystallization of starch resulting from the energetic plasma species (Thirumdas et al., 2016). In a study conducted by Lee et al. (2019), it was also investigated that the thermal properties of the rice samples were affected by treating them with cold plasma. In accordance with our findings, it was reported that the peak temperature decreased with an increase in plasma power and treatment time in basmati rice flour (Thirumdas et al., 2016; Zhang et al., 2015). A similar decrease was also reported in the gelatinization temperature of potato starch after the atmospheric nitrogen plasma treatment, which can be due to the depolymerization or change in amylose and amylopectin ratios of starch granules resulting from reactive species produced by plasma treatment. The decrease in enthalpy (ΔH) was observed in this study and can be justified by the decreased crystallinity of starch after the plasma treatment. Wang et al. (2010) reported that the ΔH shows the loss of the double helical structure of the starch molecules. The decreased enthalpy shows that the cold plasma-treated samples consume less energy for gelatinization. Moreover, it was reported that the different observations and trends in the thermal properties of various samples treated with cold plasma could be due to the fact that the starch can be degraded or cross-linked depending on the plasma treatment conditions (Sarangapani et al., 2016).

**Table 5:** The DSC parameters of different rice samples treated and untreated by PALs.

Sample	T_O_ (°C)	T_p_ (°C)	T_c_ (°C)	ΔH (J/g)
P1	59.60 ± 0.28^a^	71.10 ± 0.28^a^	85.80 ± 0.28^b^	−293.65 ± 0.49^b^
P2	43.15 ± 0.35^c^	71.45 ± 0.07^a^	90.65 ± 0.49^a^	−597.75 ± 3.18^a^
P3	34.75 ± 1.34^d^	54.15 ± 0.35^c^	45.15 ± 0.63^d^	−211.05 ± 2.05^c^
P4	49.20 ± 2.54^b^	68.30 ± 0.14^b^	89.20 ± 0.42^a^	−29.11 ± 1.68^e^
P5	16.45 ± 1.06^e^	41.90 ± 0.85^d^	63.35 ± 0.35^c^	−134.45 ± 2.47^d^

Results are presented as a mean value ± standard deviation (n = 3). Means with different letters in the same column are significantly different (*p* *<* *0.05*). (P1: Control (deionized water); P2: PAW (7.5 min); P3: PAW (15 min); P4: PAS (7.5 min); P5: PAS (15 min)). Abbreviations: PALs: plasma-activated liquids; PAW: plasma-activated water; PAS: plasma-activated saline.

### 3.5 Color parameters

The color of the rice samples as an important external factor for consumer acceptance was evaluated, and the results are shown in Table 6 (Lee et al., 2015). No great difference was observed for L* in all samples. About a*, the results for the samples treated in PAL were slightly lower than the control sample. Also, the b* slightly decreased for the samples treated in PALs than the control sample. The samples treated with 15-min PALs also had a lower b* compared to the samples with 7.5-min PALs (*p* *<* *0.05*). Therefore, it seems that the plasma treatment resulted in a decrease in b*, indicating lower yellowness for the samples. The results also showed that the treatment of the rice samples with PALs decreased the BI and increased the WI of the samples, indicating a lower browning for the PALs-treated counterpart compared to the control sample. The total color differences (ΔE) of all samples were lower than 3. According to the study conducted by Ahangari et al. (2021), the total color differences are considered very distinct (ΔE > 3), distinct (1.5 < ΔE < 3), and small differences (ΔE < 1.5). The lowest ΔE was observed in sample P2 (1.84), and the highest ΔE belongs to sample P5 (3.01). Also, the results indicated that PAS samples had a higher ΔE than PAW samples. Therefore, it can be concluded that the treatment of the rice samples with PALs on total color differences was not distinct in this study. In accordance with these results, it was reported that the color parameters of the parboiled rice were affected through treatment with low-pressure cold plasma (Sarangapani et al., 2015; Wang et al., 2024). Another study reported that the L*, a*, and b* of beef were affected by thawing with PAW attributed to the fact that the plasma activation of active substances in water can influence color substances and also can induce some reactions such as lipid oxidation which finally results in a color change (Wang et al., 2024).

**Table 6:** The color parameters of different rice samples treated and untreated by PALs, including L*, a*, b*, ΔE, whiteness index (WI), and browning index (BI).

Sample	L*	a*	b*	ΔE	WI	BI
P1	79.86 ± 0.02^a^	−0.25 ± 0.01^a^	16.67 ± 0.03^a^	0.00 ± 0.00^e^	73.85 ± 0.01^e^	1.93 ± 0.01^a^
P2	78.96 ± 0.03^d^	−0.43 ± 0.00^b^	15.07 ± 0.01^b^	1.84 ± 0.00^d^	74.12 ± 0.04^d^	1.59 ± 0.00^b^
P3	78.99 ± 0.02^cd^	−0.97 ± 0.00^e^	14.63 ± 0.04^c^	2.33 ± 0.04^c^	74.38 ± 0.01^c^	1.03 ± 0.01^c^
P4	79.10 ± 0.02^c^	−0.91 ± 0.00^d^	14.09 ± 0.04^d^	2.77 ± 0.04^b^	74.78 ± 0.01^b^	1.02 ± 0.00^c^
P5	79.58 ± 0.02^b^	−0.85 ± 0.00^c^	13.73 ± 0.07^e^	3.01 ± 0.00^a^	75.38 ± 0.02^a^	1.02 ± 0.00^c^

Results are presented as a mean value ± standard deviation (n = 3). Means with different letters in the same column are significantly different (*p* *<* *0.05*). (P1: Control (deionized water); P2: PAW (7.5 min); P3: PAW (15 min); P4: PAS (7.5 min); P5: PAS (15 min)). Abbreviations: PALs: plasma-activated liquids; PAW: plasma-activated water; PAS: plasma-activated saline.

### 3.6 Rice microstructure

**Fig. 2:**
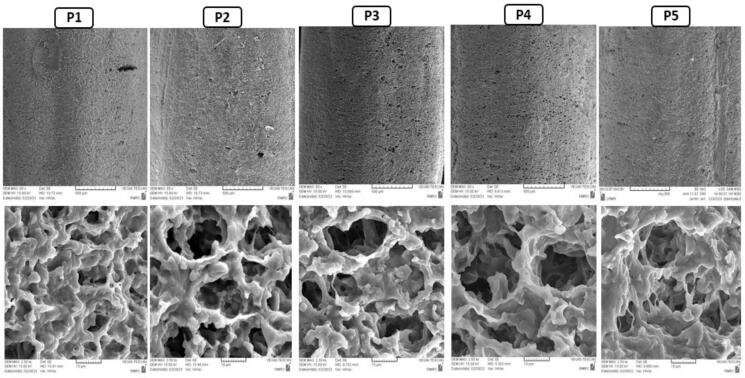
The SEM micrographs for different rice samples (P1-P5) with 80× (upper panel) and 2500 × magnification (lower panel). (P1: Control (deionized water); P2: PAW (7.5 min); P3: PAW (15 min); P4: PAS (7.5 min); P5: PAS (15 min)). Abbreviations: PAW: plasma-activated water; PAS: plasma-activated saline.

The microstructure of different rice samples was imaged using SEM, and the resulting micrographs are shown in Fig. 2. The SEM results showed that the microstructure of the control sample was denser than the samples soaked in PALs. Therefore, the results indicated that the densest microstructure was related to the P1 sample as a control sample. Higher numbers of holes were observed on the surfaces of the samples soaked in the PALs than in the control sample, which was soaked in non-treated deionized water. It can be seen that the microstructure of the samples soaked in the PALs was more porous and showed larger hollow cavities inside the grains. Moreover, according to the results, it seems that the plasma treatment time of water (7.5 and 15 min) had no significant effect on the rice microstructure. No significant differences were also observed on the microstructure of the rice samples soaked in PAW and PAS. In accordance with these findings, Chen et al. (2012) reported that the rice bran lost its natural morphology by applying plasma treatment. Thirumdas et al. (2015) reported that the plasma treatment altered the natural morphology of the rice grain and resulted in the formation of holes in the grain surfaces, which can increase water penetration.

## 4 Conclusions

In the present study, PAW and PAS were used to reduce the heavy metal content in rice. After that, the effect of PALs on the texture, thermal properties, and microstructure of rice was also investigated. The results showed that the soaking of rice in PALs resulted in a significant decrease in arsenic, cadmium, and lead contents in the rice samples. The TPA analysis also showed a softer texture for the samples soaked in the PALs compared to the control sample. The DSC results indicated that the soaking of rice in PALs resulted in a decrease in its starch gelatinization temperature. The color parameters and the microstructural features of the rice samples were also significantly affected by treatment with PALs. Generally, the results of this study suggested that the soaking of rice in PALs can be used as an efficient strategy to reduce the amount of its heavy metals and can also be considered as a method to modify the textural and thermal properties of rice to produce a sample with a softer texture requiring a lower cooking time. However, it seems that more studies are needed to investigate the effect of plasma-activated liquids on rice properties, especially its nutritional value and safety parameters, as well as to present a more specific explanation for the mechanism of PALs in the reduction of heavy metals in food products.

## CRediT authorship contribution statement

**Shahnaz Bohlooli:** Writing – review & editing, Writing – original draft, Validation, Software, Investigation, Data curation. **Yousef Ramezan:** Writing – review & editing, Writing – original draft, Validation, Supervision, Project administration, Data curation, Conceptualization. **Fatemeh Esfarjani:** Resources, Project administration, Conceptualization. **Hedayat Hosseini:** Resources, Methodology. **Soheyl Eskandari:** Resources, Methodology.

## Declaration of competing interest

The authors declare that they have no known competing financial interests or personal relationships that could have appeared to influence the work reported in this paper.

## Data Availability

Data will be made available on request.
